# Supplementary material for: Direct and indirect pathways for heterosynaptic interaction underlying developmental synapse elimination in the mouse cerebellum
Source: Commun Biol. 2024 Jul 3;7:806. doi: 10.1038/s42003-024-06447-4 (PMC11222442; doi:10.1038/s42003-024-06447-4)
Supplement: Supplementary file 2 — Supplementary Information [file 42003_2024_6447_MOESM2_ESM.pdf]

# Supplementary Figures

# Direct and indirect pathways for heterosynaptic interaction underlying developmental synapse elimination in cerebellar Purkinje cells

Hisako Nakayama, Taisuke Miyazaki, Manabu Abe, Maya Yamazaki, Yoshinobu Kawamura, Myeongjeong Choo, Kohtarou Konno, Shinya Kawata, Naofumi Uesaka, Kouichi Hashimoto, Mariko Miyata, Kenji Sakimura, Masahiko Watanabe & Masanobu Kano

# Supplementary Figure 1

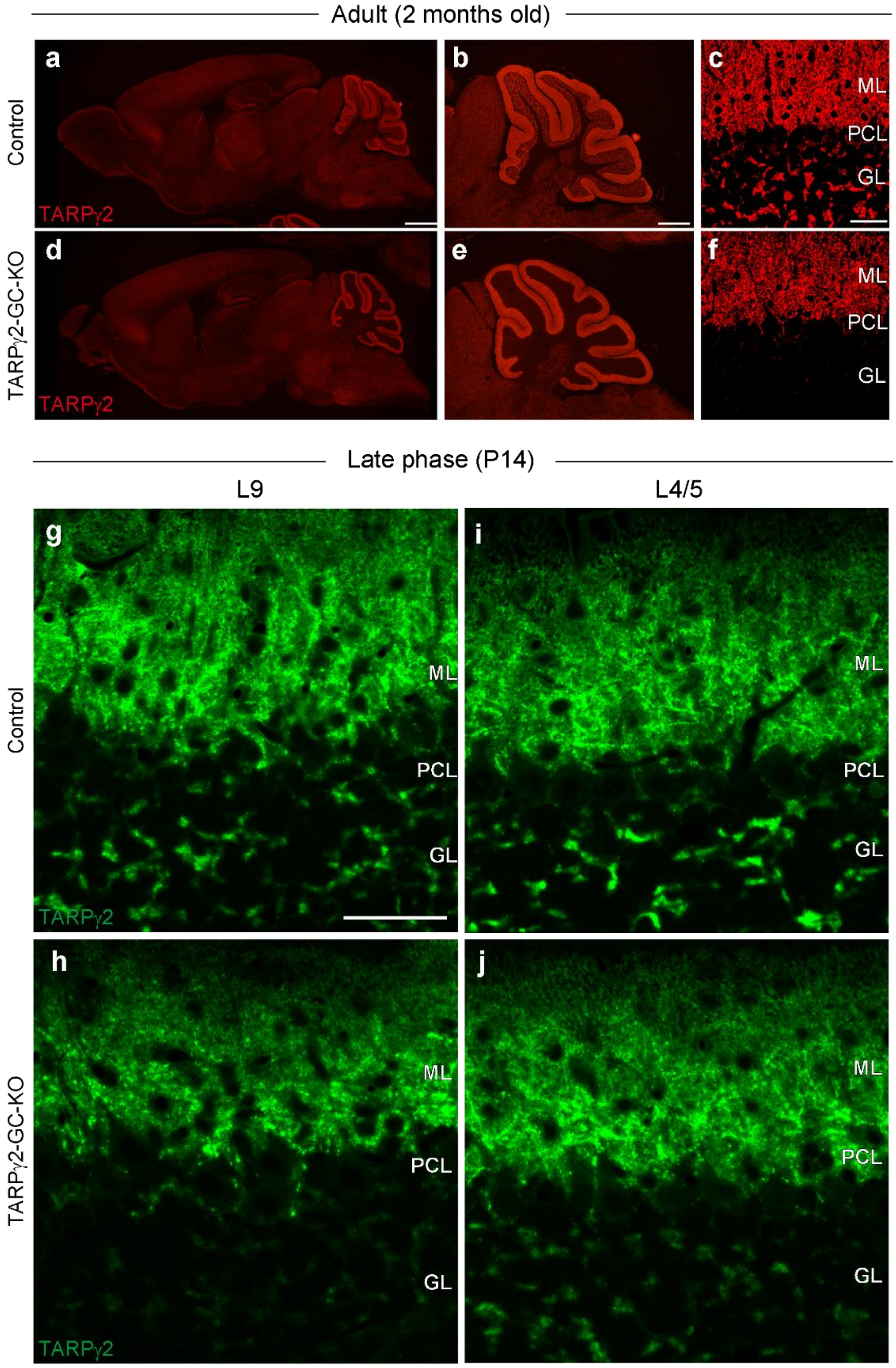

**Supplementary Figure 1. Deficient TARPγ2 expression in GCs in lobules 8-9 but not in lobules 1-4/5 of TARPγ2-GC KO mice during the third postnatal week**

**a-f** Immunostaining for TARPγ2 in a mature control (**a-c**) and a mature TARPγ2-GC-KO (**d-f**) mouse at 2 months of age showing the TARPγ2 expression in the whole brain (**a, d**), the whole cerebellum (**b, e**), and the three cerebellar cortical layers (**c, f**). ML: molecular layer, PCL: Purkinje cell layer, GL: granule cell layer. Scale bar: 1000 μm for **a**, 200 μm for **b,** and 50 μm for **c**.

**g-j** Immunostaining for TARPγ2 in lobule 9 (**g, h**) and lobule 4/5 (**i, j**) of the cerebellum from a control (**g, i**) and a TARPγ2-GC-KO (**h, j**) mice at P14. Scale bar, 50 μm

## Supplementary Figure 2

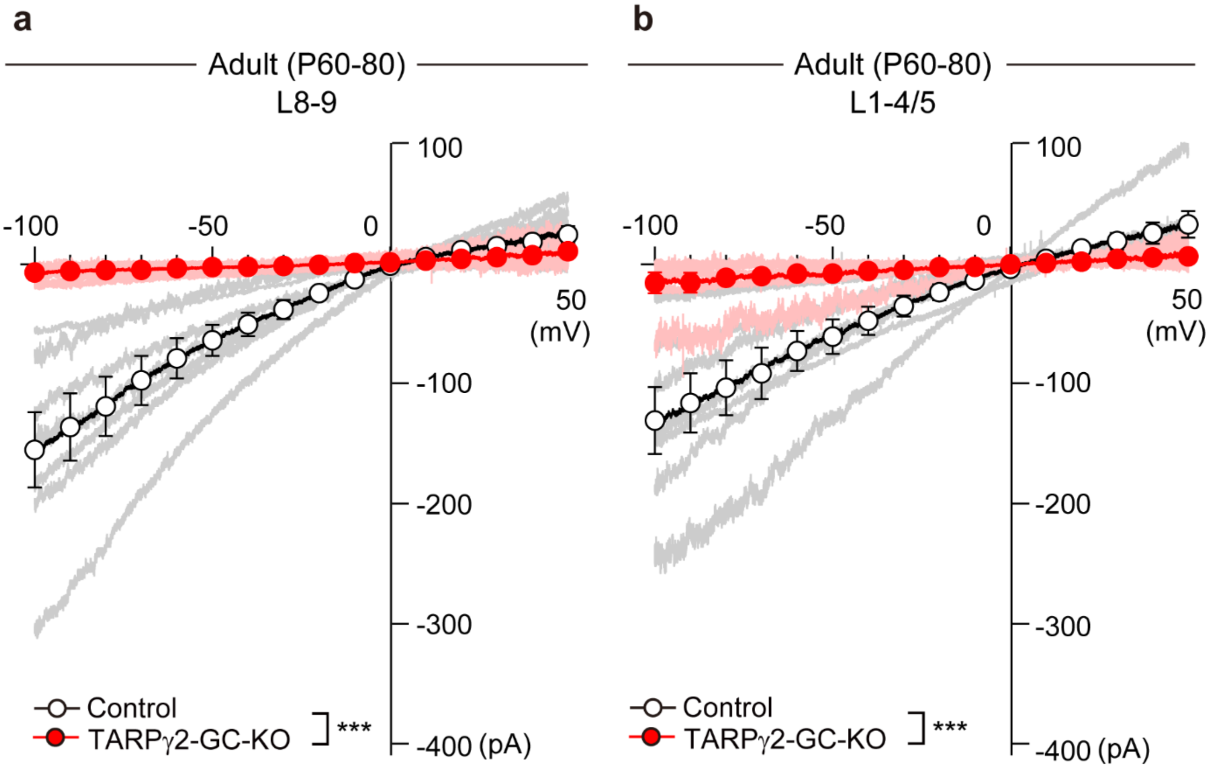


**Supplementary Figure 2. Deficient AMPAR-mediated currents in GCs in both lobules 8-9 and lobules 1-4/5 of mature TARPγ2-GC-KO mice**

**a, b** Instantaneous I–V relationships of the AMPA (10 μM)-induced current evoked in GCs of lobules 8-9 (**a**) and lobules 1-4/5 (**b**) of the cerebellum from control (white symbols with bold black line,, n = 7 for **a** and **b**) and TARPγ2-GC-KO (red symbols with red bold line,, n = 8 for **a** and **b**) mice from P60 to P80. Data from individual cells are shown in faint lines. $P < 0.001$ for **a** and **b**, Two-way Repeated-Measures ANOVA. Data are mean ± SEM.

## Supplementary Figure 3

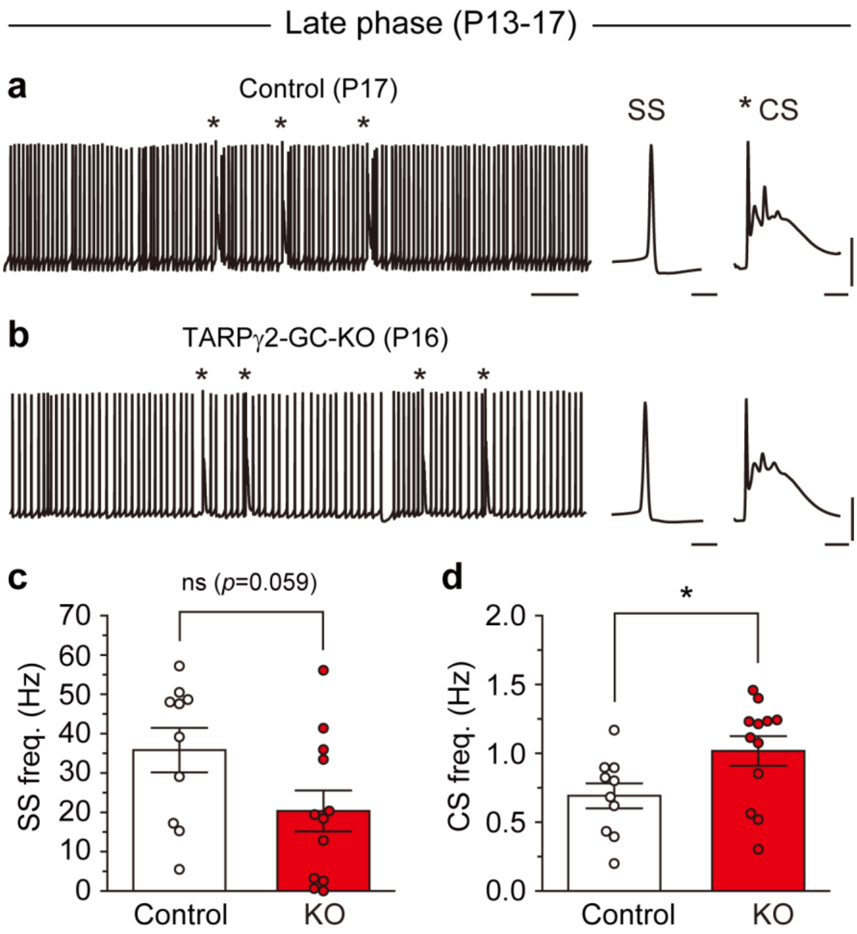


**Supplementary Figure 3. Spontaneous firing and synaptic potentials in PCs recorded from control and TARPγ2-GC-KO mice *in vivo***

**a, b** Sample traces of membrane potentials in PCs from a P17 control (**a**) and a P16 TARPγ2-GC-KO (**b**) mouse. SS: simple spike, CS: complex spike. The resting membrane potential was -43 mV for **a** and -44 mV for **b**. Horizontal scale bars, 0.5 s (left traces), 1 ms (SS), and 5 ms (CS). Vertical scale bar, 20 mV.

**c, d** Summary bar graphs showing the average frequency of simple spikes (**c**) and complex spikes (**d**) in control and TARPγ2-GC-KO mice at P13-P17. $P < 0.05$, *t*-test. Data are mean ± SEM.

## Supplementary Figure 4

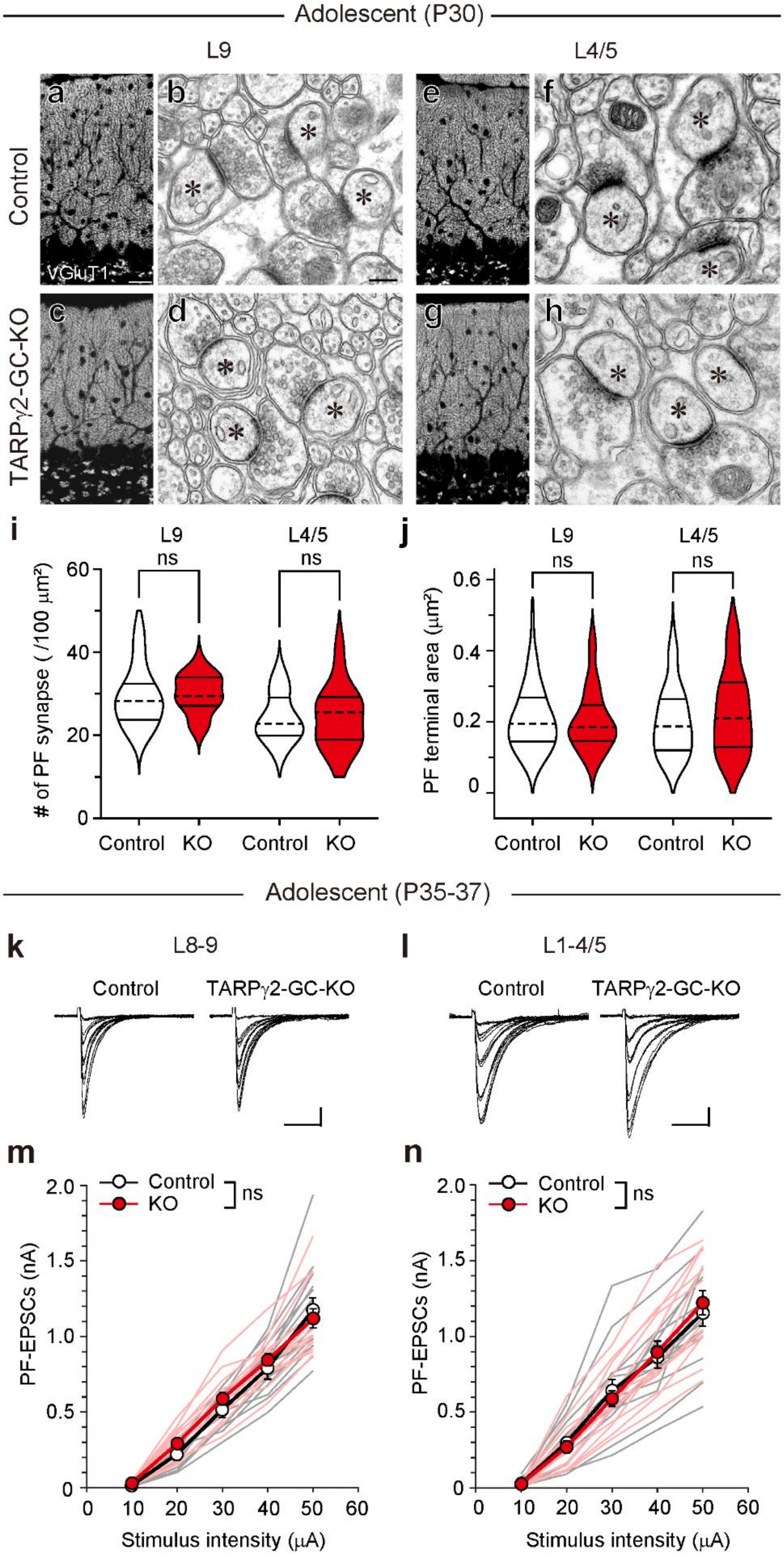

**Supplementary Figure 4. Quantitative morphological and electrophysiological analyses of PF-PC synapse in adolescent control and TARPγ2-GC-KO mice**

**a-h** Light microscopic immunofluorescent images for the PF terminal marker VGluT1 (**a, c, e, g**) and electron microscopic images showing PF-PC synapses (**b, f, d, h**) in lobule 9 (**a-d**) and lobule 4/5 (**e-h**) from control (**a, b, e, f**) and TARPγ2-GC-KO (**c, d, g, h**) mice at P30. Scale bars in **a** and **b** are 20 μm and 200 nm, respectively. Asterisks in **b, f, d, h** indicate PC spines. The distribution and the punctate staining pattern of VGluT1 signals and the morphology of PF-PC synapses appear normal in TARPγ2-GC-KO mice in both lobules 9 and 4/5.

**i, j** Summary violin plots for the density of PF-PC synapses (**i**) and the PF terminal area (**j**) in lobule 9 and lobule 4/5 from control (open plots) and TARPγ2-GC-KO (filled plots) mice. The dashed line within each violin plot represents the median, while the solid lines at the top and bottom indicate the 75th and 25th percentiles, respectively.

**k-n** Sample traces of PF-EPSCs with stepwise increase of stimulus intensity (**k, l**) and summary graphs showing the stimulus-response relationship of PF-EPSCs (**m, n**) in lobule 9 (**k, m**) and lobule 4/5 (**l, n**) of control (white symbols with bold black line, n = 13 for **m**, n = 15 for **n**) and TARPγ2-GC-KO (red symbols with red bold line,, n = 14 for **m**, n = 14 for **n**) mice at P35 to P37. Data from individual cells are shown in faint lines. No difference was noted in the stimulus-response relationship between the two mouse strains in either lobule. $P$ = 0.553 for lobule 9 and $P$ = 0.953 for lobule 4/5. Two-way Repeated-Measures ANOVA. Data are mean ± SEM.

## Supplementary Figure 5

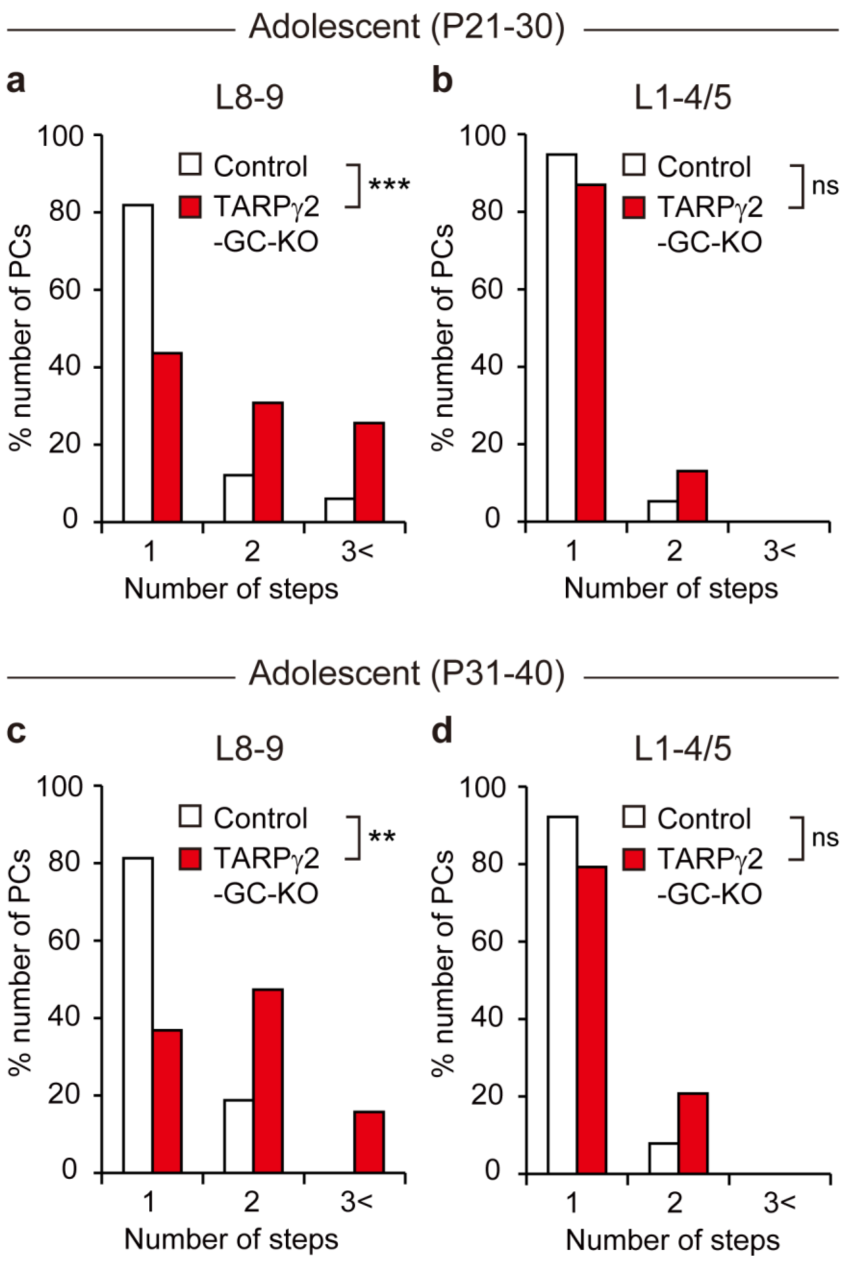


**Supplementary Figure 5**. **CF innervation of PCs from P21 to P30 and from P31 to P40**

**a-d** Persistent multiple CF innervation of PCs in cerebellar lobules 8-9 of TARPγ2-GC-KO mice from P21 to P40. Frequency distribution histograms of PCs in terms of the number of discrete CF-EPSC steps during P21-P30 (**a, b**) and P31-P40 (**c, d**) in cerebellar lobules 8-9 (**a, c**) and 1-4/5 (**b, d**) from control (white columns) and TARPγ2-GC-KO (red columns) mice. ** $P < 0.01$, *** and $P < 0.001$, Mann-Whitney *U* test.

## Supplementary Figure 6

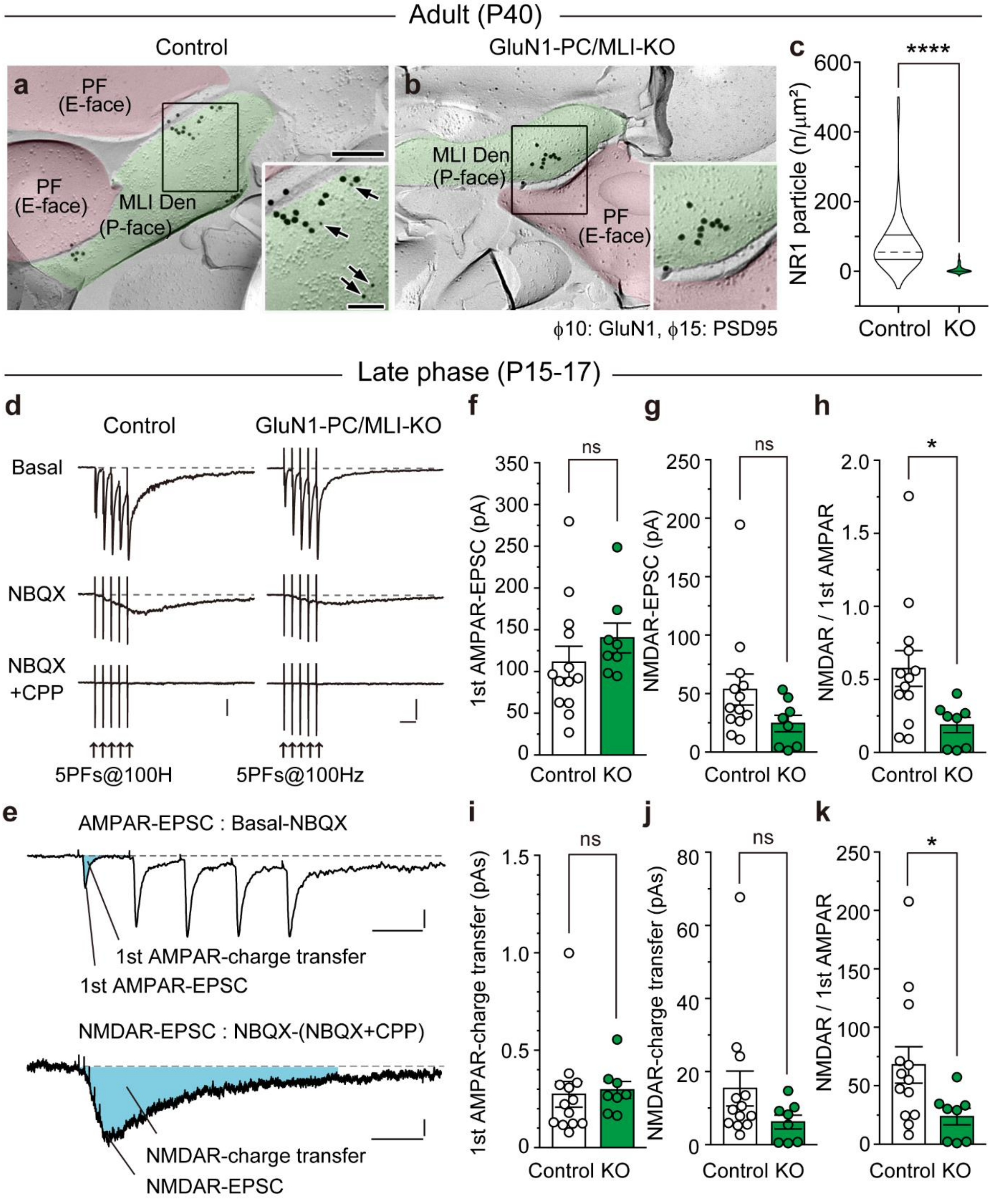


**Supplementary Figure 6. Reduction of PF-induced NMDAR responses in MLIs of GluN1-MLI/PC-KO mice**

**a, b** SDS-digested freeze-fracture replica labeling of immunogold particles for GluN1 (10 nm particles) and PSD95 (15 nm particles) in a control (**a**) and a GluN1-MLI/PC-KO (**b**) mouse at P40. Scale bar: 200 nm for low magnification images, 100 nm for

high magnification images.

**c** Summary violin plots for the number of NR1 gold particles per μm$^2$ of MLI dendrites in control (open plot) and GluN1-MLI/PC-KO (filled plot) mice. The dashed line within each violin plot represents the median, while the solid lines at the top and bottom indicate the 75th and 25th percentiles, respectively.

**d** Sample traces of EPSCs in MLIs elicited by high-frequency stimulation of PFs (5 pulses at 100 Hz) in a control (left panels) and a GluN1-MLI/PC-KO (right panels) mouse. Basal EPSCs were recorded at a holding potential of -70 mV in 0 mM $Mg^{2+}$- and 10 μM glycine-containing bath solution to enhance NMDAR-mediated currents. NBQX (10 μM) and R-CPP (5 μM) were added sequentially to the bathing solution. Scale bars, 20 ms and 50 pA.

**e** Sample traces of EPSCs in MLIs elicited by high-frequency stimulation of PFs with higher time resolution than in **d** to illustrate how to measure the peak amplitude and charge transfer of the 1$^{st}$ AMPAR component and the NMDAR component. The AMPAR-mediated EPSC components were obtained by subtracting the EPSCs in the presence of 10 μM NBQX from the basal EPSCs. The NMDAR-mediated EPSC components were obtained by subtracting EPSCs in the presence of 10 μM NBQX and 5 μM R-CPP from those with 10 μM NBQX. The areas indicated with light blue exemplify how the charge transfer of the 1$^{st}$ AMPAR-mediated EPSC (measured for 10 ms after the 1$^{st}$ stimulus) and that of NMDAR-mediated EPSCs (measured for 500 ms after the 1$^{st}$ stimulus) were measured.

**f-h** Summary bar graphs showing the peak amplitude of the 1$^{st}$ AMPAR component (**f**), that of the NMDAR component (**g**), and the ratio of the peak NMDAR component over the 1$^{st}$ AMPAR component (**h**) of EPSCs in MLIs by high-frequency PF stimulation in control (white columns) and GluN1-MLI/PC-KO (green columns) mice. (**f,** Control: 111 ± 19.0 pA, n = 13, 4 mice; GluN1-MLI/PC-KO: 140 ± 17.8 pA, n = 8, 3 mice, *P* = 0.111. **g,** Control: 53.5 ± 13.2 pA, n = 13, 4 mice; GluN1-MLI/PC-KO: 24.4 ± 7.07 pA, n = 8, 3 mice, *P* = 0.060. **h,** Control: 0.57 ± 0.12, n = 13, 4 mice; GluN1-MLI/PC-KO: 0.19 ± 0.05, n = 8, 3 mice, **P* = 0.01. Mann-Whitney *U* test). Data are mean ± SEM.

**i-k** Summary bar graphs showing the charge transfer of the 1$^{st}$ AMPAR component (**i**), that of the NMDAR component (**j**), and the ratio of the charge transfer of the NMDAR component over that of the 1$^{st}$ AMPAR component (**k**) in control (open columns) and GluN1-MLI/PC-KO (filled columns) mice. (**i,** Control: 0.27 ± 0.07 pAs, n = 13, 4 mice; GluN1-MLI/PC-KO: 0.30 ± 0.04 pAs, n = 8, 3 mice, *P* = 0.277. **j,** Control: 15.3 ± 4.79 pAs, n = 13, 4 mice; GluN1-MLI/PC-KO: 6.16 ± 1.88 pAs, n = 8, 3 mice, *P* = 0.128. **k,** Control: 67.9 ± 15.6, n = 13, 4 mice; GluN1-MLI/PC-KO: 23.7 ± 7.15, n = 8, 3 mice, **P* = 0.036. Mann-Whitney *U* test). Data are mean ± SEM.

**Supplementary Figure 7**

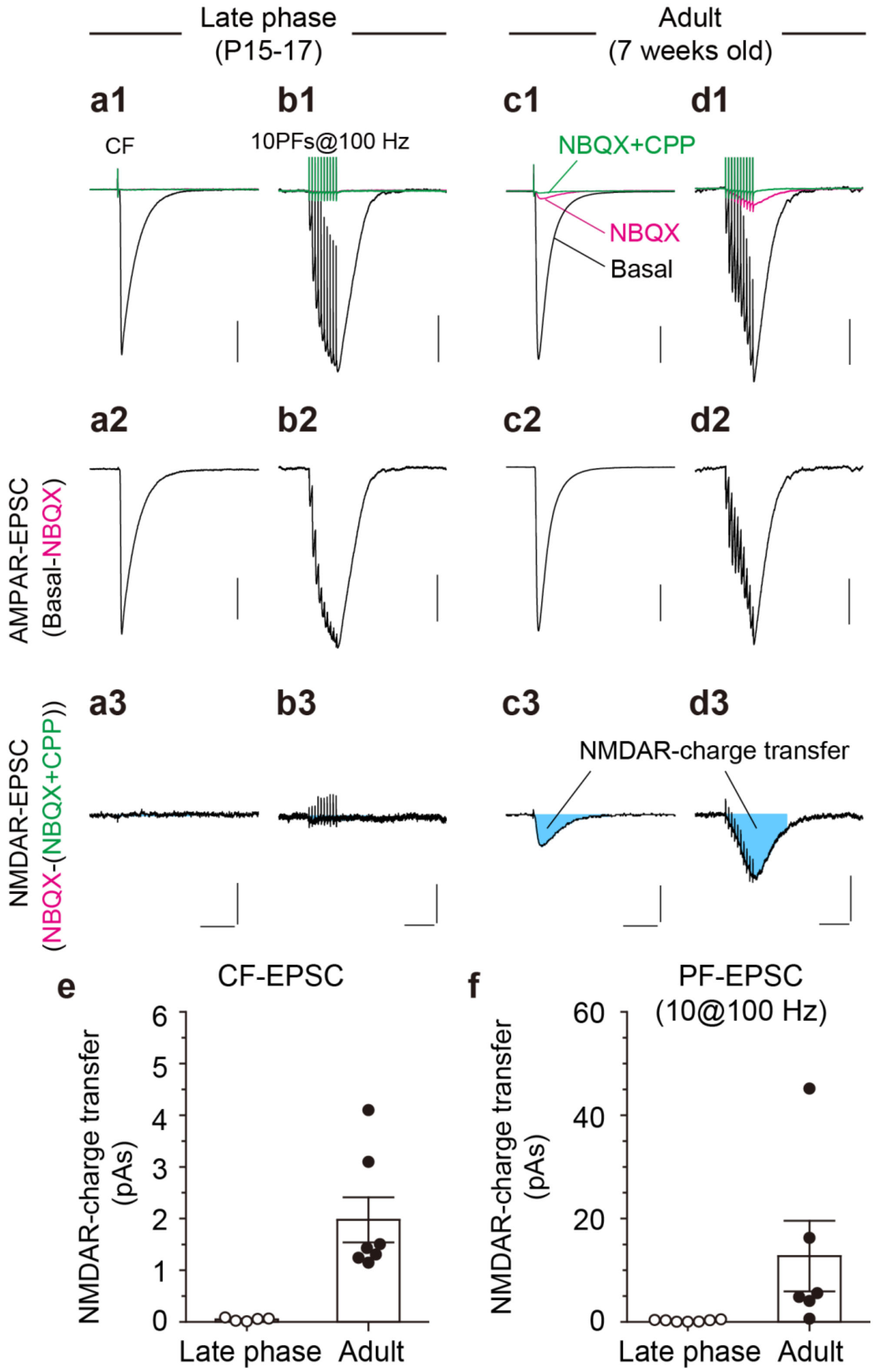

**Supplementary Figure 7. Deficient NMDAR-mediated component in CF-EPSCs and PF-EPSCs of PCs in P17 mice**

**a-d** Sample traces of CF-EPSCs (**a1-3, c1-3**) and PF-EPSCs in response to high-frequency PF stimulation of 10 pulses at 100 Hz (**b1-3, d1-3**) recorded in a PC of a control mouse at P16 (**a1-3, b1-3**) and 7 weeks of age (**c1-3, d1-3**). Holding potential, -70 mV. Scale bars for CF-EPSCs, 20 ms (**a1, a2, c1, c2**) and 200 pA (**a3, c3**). Scale bars for PF-EPSCs, 100 ms, 300 pA (**b1, b2**), 50 pA (**b3**), 500 pA (**d1, d2**) and 100 pA (**d3**). The basal EPSCs were recorded in a bathing solution containing no $Mg^{2+}$ and 10 μM glycine to maximize NMDAR activation. In addition, we included in the bathing solution 100 μM CPCCOEt (Tocris) to block mGlu1, picrotoxin (100 μM) to block $GABA_A$Rs, and 0.3 μM NBQX to partially block AMPARs and improve the accuracy of voltage clamp for recording CF-EPSCs at the holding potential of -70 mV. After recording basal CF-EPSCs or PF-EPSCs, NBQX (10 μM) and R-CPP (5 μM) were added sequentially to the bathing solution. The AMPAR-mediated EPSC components (AMPAR_EPSC, **a2, b2, c2, d2**) were obtained by subtracting the EPSCs in the presence of 5 μM NBQX from the basal EPSCs. The NMDAR-mediated EPSC components (NMDAR_EPSC, **a3, b3, c3, d3**) were obtained by subtracting EPSCs in the presence of 10 μM NBQX and 5 μM R-CPP from those with 10 μM NBQX. The areas indicated with light blue in **c3** and **d3** exemplify how charge transfers of NMDAR-mediated components were measured in **e** and **f**.

**e, f,** Summary bar graphs showing the NMDAR-mediated charge transfer of CF-EPSCs (**e**, measured for 50 ms after the CF stimulus. Late phase: 0.05 ± 0.01 pAs, n = 5, 2 mice; Adult: 1.97 ± 0.44 pAs, n = 7, 2 mice) and PF-EPSCs (**f**, measured for 200 ms after the 1$^{st}$ PF stimulus. Late phase: 0.25 ± 0.07 pAs, n = 6, 2 mice; Adult: 12.7 ± 6.83 pAs, n = 6, 2 mice). Data are mean ± SEM.
