# Supplementary material for: Direct and indirect pathways for heterosynaptic interaction underlying developmental synapse elimination in the mouse cerebellum
Source: Commun Biol. 2024 Jul 3;7:806. doi: 10.1038/s42003-024-06447-4 (PMC11222442; doi:10.1038/s42003-024-06447-4)
Supplement: Supplementary file 5 — Reporting Summary [file 42003_2024_6447_MOESM5_ESM.pdf]

nature portfolio

Corresponding author(s): Masanobu Kano

Last updated by author(s): May 31, 2024

Reporting Summary

Nature Portfolio wishes to improve the reproducibility of the work that we publish. This form provides structure for consistency and transparency in reporting. For further information on Nature Portfolio policies, see our Editorial Policies and the Editorial Policy Checklist.

Statistics

For all statistical analyses, confirm that the following items are present in the figure legend, table legend, main text, or Methods section.

n/a	Confirmed	
☐	☒	The exact sample size (*n*) for each experimental group/condition, given as a discrete number and unit of measurement
☐	☒	A statement on whether measurements were taken from distinct samples or whether the same sample was measured repeatedly
☐	☒	The statistical test(s) used AND whether they are one- or two-sided<br>*Only common tests should be described solely by name; describe more complex techniques in the Methods section.*
☐	☒	A description of all covariates tested
☐	☒	A description of any assumptions or corrections, such as tests of normality and adjustment for multiple comparisons
☐	☒	A full description of the statistical parameters including central tendency (e.g. means) or other basic estimates (e.g. regression coefficient) AND variation (e.g. standard deviation) or associated estimates of uncertainty (e.g. confidence intervals)
☐	☒	For null hypothesis testing, the test statistic (e.g. *F*, *t*, *r*) with confidence intervals, effect sizes, degrees of freedom and *P* value noted<br>*Give P values as exact values whenever suitable.*
☒	☐	For Bayesian analysis, information on the choice of priors and Markov chain Monte Carlo settings
☒	☐	For hierarchical and complex designs, identification of the appropriate level for tests and full reporting of outcomes
☒	☐	Estimates of effect sizes (e.g. Cohen's *d*, Pearson's *r*), indicating how they were calculated

Our web collection on statistics for biologists contains articles on many of the points above.

Software and code

Policy information about availability of computer code

Data collection	Immunohistochemistry data were acquired using MetaMorph (Molecular Devices). Electrophysiology data were acquired using Pulse and Patchmaster (HEKA Elektronik), and Axograph X (Axograph Scientific).
Data analysis	Graph pad prism version9, Fit Master (HEKA Elektronic), Sigma Plot 14.5 (SYSTAT), Axogragh X (Axograph Scientific), Excel (Mirosoft), Origin Pro (Origin Lab)

For manuscripts utilizing custom algorithms or software that are central to the research but not yet described in published literature, software must be made available to editors and reviewers. We strongly encourage code deposition in a community repository (e.g. GitHub). See the Nature Portfolio guidelines for submitting code & software for further information.

Data

Policy information about availability of data

All manuscripts must include a data availability statement. This statement should provide the following information, where applicable:

- Accession codes, unique identifiers, or web links for publicly available datasets
- A description of any restrictions on data availability
- For clinical datasets or third party data, please ensure that the statement adheres to our policy

The datasets in the current study are available from the corresponding author upon reasonable request.

Research involving human participants, their data, or biological material

Policy information about studies with human participants or human data. See also policy information about sex, gender (identity/presentation), and sexual orientation and race, ethnicity and racism.

Reporting on sex and gender	N/A
Reporting on race, ethnicity, or other socially relevant groupings	N/A
Population characteristics	N/A
Recruitment	N/A
Ethics oversight	N/A

Note that full information on the approval of the study protocol must also be provided in the manuscript.

Field-specific reporting

Please select the one below that is the best fit for your research. If you are not sure, read the appropriate sections before making your selection.

☒ Life sciences ☐ Behavioural & social sciences ☐ Ecological, evolutionary & environmental sciences

For a reference copy of the document with all sections, see nature.com/documents/nr-reporting-summary-flat.pdf

Life sciences study design

All studies must disclose on these points even when the disclosure is negative.

Sample size	The sample size was estimated based on previous reports. We conducted the experiments using more than 3 samples per group.
Data exclusions	Electrophysiological experiments were not analysed if a noisy recording condition was present.
Replication	The electrophysiological data of the control group are reproducible across experiments (Fig. 2, 4, 5, and 6).<br>Also, our data are consistent with the results of several previous studies:<br>Effects of TARPg2 KO on the expression and function of AMPARs (Fig. 1 and Supplementary Fig. 2).<br>Jackson and Nicoll. Stargazin (TARP gamma-2) is required for compartment-specific AMPA receptor trafficking and synaptic plasticity in cerebellar stellate cells. J Neurosci. 31(11):3939-52 (2011)<br>Hashimoto et al. Impairment of AMPA receptor function in cerebellar granule cells of ataxic mutant mouse stargazer. J Neurosci. 19(14):6027-36 (1999)<br><br>Effects of mGluR1 KD in Purkinje cell on the climbing fiber synapse elimination (Fig. 4).<br>Choo et al. Retrograde BDNF to TrkB signaling promotes synapse elimination in the developing cerebellum. Nat Commun. 8(1):195 (2017)<br>Uesaka et al. Retrograde semaphorin signaling regulates synapse elimination in the developing mouse brain. Science. 344(6187):1020-3 (2014)<br>Kano et al. Persistent multiple climbing fiber innervation of cerebellar Purkinje cells in mice lacking mGluR1. Neuron. 18(1):71-9 (1997)<br><br>Contribution of NMDARs to EPSCs in Purkinje cells (Supplementary Fig. 7).<br>Piochon et al. NMDA receptor contribution to the climbing fiber response in the adult mouse Purkinje cell. J Neurosci. 27(40):10797-809 (2007)<br>Renzi et al. Climbing-fiber activation of NMDA receptors in Purkinje cells of adult mice. J Physiol. 585(Pt 1):91-101 (2007)<br>Llano et al. Synaptic- and agonist-induced excitatory currents of Purkinje cells in rat cerebellar slices. J Physiol. 434:183-213 (1991)<br>Konnerth et al. Synaptic currents in cerebellar Purkinje cells. Proc Natl Acad Sci U S A. 87(7):2662-5 (1990)
Randomization	To randomize the rearing conditions, experiments were conducted using littermate mice and data were allocated into experimental groups.
Blinding	The acquisition and analysis of the electrophysiological data were performed blindly.

Reporting for specific materials, systems and methods

We require information from authors about some types of materials, experimental systems and methods used in many studies. Here, indicate whether each material, system or method listed is relevant to your study. If you are not sure if a list item applies to your research, read the appropriate section before selecting a response.

Materials & experimental systems

n/a	Involved in the study	
☐	☒	Antibodies
☒	☐	Eukaryotic cell lines
☒	☐	Palaeontology and archaeology
☐	☒	Animals and other organisms
☒	☐	Clinical data
☒	☐	Dual use research of concern
☒	☐	Plants

Methods

n/a	Involved in the study	
☒	☐	ChIP-seq
☒	☐	Flow cytometry
☒	☐	MRI-based neuroimaging

Antibodies

Antibodies used

rabbit anti-GluA2 (847-863aa; RRID, AB_2571754) MSFR102320, Nittobo.
rabbit anti-TARPγ2 (302-318aa; RRID, AB_2571844) MSFR105740, Nittobo.
goat anti-calbindin (RRID, AB_2532104) MSFR100400, Nittobo.
guinea pig anti-VGluT2 (RRID, AB_2341096) MSFR106280, Nittobo.
guinea pig anti-VGluT1 (RRID, AB_2571618) MSFR106180, Nittobo.
rabbit anti-dextran Alexa488 (DA488, Invitrogen) A-11094, Invtrogen.
These six antibodies described above were used for immunohistochemistry.
rabbit anti-GluN1 antibody (RRID, AB_2571604) MSFR102670, Nittobo.
guinea-pig anti-PSD95 antibody (RRID, AB_2571612) MSFR105180, Nittobo.
These two antibodies described above were used for SDS-FRL.

Validation

All antibodies used in the study are validated in the manufacturer's website in NIttobo (https://nittobo-nmd.co.jp/english/product/product_list.php) and Invitrogen (https://www.thermofisher.com/antibody/product/Alexa-Fluor-488-Antibody-Polyclonal/A-11094).

Animals and other research organisms

Policy information about studies involving animals; ARRIVE guidelines recommended for reporting animal research, and Sex and Gender in Research

Laboratory animals

Both male and female C57BL/6NCrSlc mice (Japan SLC, Inc, RRID: MGI:5295404), TARPg2(flox/flox); GluN2C(cre/wt) mice, GluN1(flox/flox); GluN2C(cre/wt) mice, and GluN1(flox/flox); GluD2(cre/wt) mice aged P6-P80 were used.

Wild animals

No wild animals were used in this study.

Reporting on sex

We used both sexes of mice.

Field-collected samples

No field-collected samples were used in this study.

Ethics oversight

All experiments were conducted according to the guidelines of the experimental animal ethics committees and the biosafety committee for living modified organisms of The University of Tokyo, Tokyo Women's Medical University, Hiroshima University, Niigata University, Hokkaido University, and the Japan Neuroscience Society.

Note that full information on the approval of the study protocol must also be provided in the manuscript.

Plants

Seed stocks

N/A

Novel plant genotypes

N/A

Authentication

N/A
